# A Remote Palm Domain Residue of RB69 DNA Polymerase Is Critical for Enzyme Activity and Influences the Conformation of the Active Site

**DOI:** 10.1371/journal.pone.0076700

**Published:** 2013-10-07

**Authors:** Agata Jacewicz, Anna Trzemecka, Kip E. Guja, Danuta Plochocka, Elena Yakubovskaya, Anna Bebenek, Miguel Garcia-Diaz

**Affiliations:** 1 Department of Molecular Biology, Institute of Biochemistry and Biophysics, Polish Academy of Sciences, Warsaw, Poland; 2 Department of Bioinformatics, Institute of Biochemistry and Biophysics, Polish Academy of Sciences, Warsaw, Poland; 3 Department of Pharmacological Sciences, Stony Brook University, Stony Brook, New York, United States of America; Centro de Biología Molecular Severo Ochoa (CSIC-UAM), Spain

## Abstract

Non-conserved amino acids that are far removed from the active site can sometimes have an unexpected effect on enzyme catalysis. We have investigated the effects of alanine replacement of residues distant from the active site of the replicative RB69 DNA polymerase, and identified a substitution in a weakly conserved palm residue (D714A), that renders the enzyme incapable of sustaining phage replication *in vivo*. D714, located several angstroms away from the active site, does not contact the DNA or the incoming dNTP, and our apoenzyme and ternary crystal structures of the Pol^D714A^ mutant demonstrate that D714A does not affect the overall structure of the protein. The structures reveal a conformational change of several amino acid side chains, which cascade out from the site of the substitution towards the catalytic center, substantially perturbing the geometry of the active site. Consistent with these structural observations, the mutant has a significantly reduced k_*pol*_ for correct incorporation. We propose that the observed structural changes underlie the severe polymerization defect and thus D714 is a remote, non-catalytic residue that is nevertheless critical for maintaining an optimal active site conformation. This represents a striking example of an action-at-a-distance interaction.

## Introduction

The gp43 DNA polymerase of the RB69 bacteriophage (RB69 Pol) bears primary responsibility for the faithful and efficient replication of the viral genome. RB69 Pol belongs to the B-class of DNA polymerases and is closely related to T4 phage DNA polymerase. It shares significant sequence similarity with eukaryotic replicases of the same family, such as the α, δ and ε polymerases, which carry out the bulk of chromosomal replication in higher organisms [1,2]. Several crystal structures of RB69 Pol alone [3], as well as in complex with DNA and the incoming nucleotide [4–6] in many different conformations are available to date, enabling extensive structure–function studies of this model replicative polymerase.

RB69 DNA Pol synthetizes DNA with high accuracy, introducing one mutation per 2 x 10^8^ replicated bases [1,7]. This high fidelity is achieved through the combined action of its polymerase (Pol) and 3′-5′-exonuclease (Exo) activities, which enable stringent selection of the correct incoming nucleotide and excision of the mismatches resulting from misincorporations. Binding of the correct nucleotide induces large conformational changes in the enzyme, manifested by movement of the fingers subdomain towards the catalytic center (palm subdomain), and the formation of a tight binding pocket for the nascent base pair. The newly formed pocket preferentially accommodates correct nucleotides, whereas mispair geometry nearly always triggers reopening of the fingers and the release of the mismatched dNTP [8].

Numerous biochemical and structural studies have focused on the active site of RB69 DNA Pol and provided information about the function of several highly conserved residues [9-14]. These include immediate neighbors of the catalytic aspartate triad D411, D621 and D623, engaged directly or indirectly in binding the divalent metal ion and the 3′–end of the primer, as well as several fingers subdomain residues constituting the dNTP binding site and responsible for mismatch discrimination. In addition, several other studies focused on RB69 Pol residues that control the fidelity of DNA replication *via* hydrogen bonding with the minor groove of the primer-template duplex and specifically recognize the geometry of Watson-Crick base pairing up to 4 nucleotides upstream of the active site [5,6].

**Figure 1:**
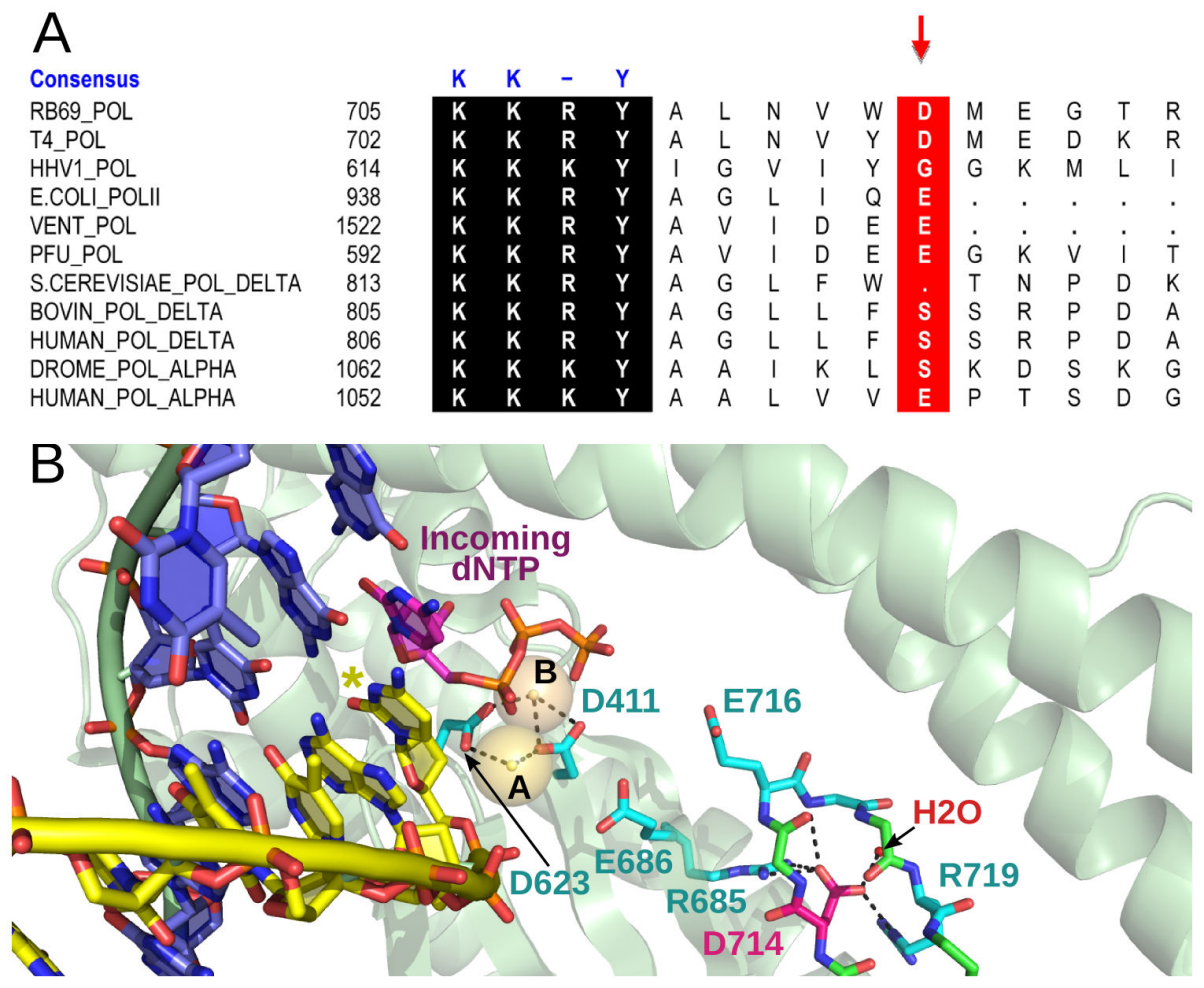
Conservation and molecular interactions of the D714 residue. (**A**) Sequence alignment of DNA polymerases from the B-family. D714 of RB69 DNA Pol and the corresponding residues in homologous DNA polymerases are indicated with a red arrow. The strongly conserved KKRY motif, responsible for binding of DNA in B-conformation, is shown in a black box. Aligned sequences include RB69 DNA Pol (RB69_POL; U34036), T4 DNA polymerase (T4_POL; M10160), human herpes virus 1 DNA polymerase (HHV1_Pol; EU704683), DNA polymerase from *Thermococcus literalis* (VENT_Pol; M74198), DNA Pol II from *Escherichia coli* (*E.COLI*_POLII; X54847), DNA polymerase from *Pyrococcus furiosus* (PFU_POL; D12983), *Saccharomyces cerevisiae* polymerase δ (S.CEREVISAE_POL_DELTA; X15477), *Bos taurus* polymerase δ (BOVINE_POL_DELTA; M80395), human polymerase δ (HUMAN_POL_DELTA; M80397), *Drosophila melanogaster* polymerase α (DROME_POL_DELTA; D90310) and human polymerase α (HUMAN_POL_ALPHA; X06745). The alignment was based on the sequence comparison published by Braithwaite and Ito [17]. (**B**) Cartoon of the active center of RB69 DNA polymerase in complex with the DNA duplex (primer strand – yellow, template strand – navy blue) and the incoming dNTP (violet and orange), illustrating the hydrogen-bonding interactions established by D714. D714 is shown in magenta. Hydrogen bonds are represented as black dashed lines. The side chains of M715 and T718 were removed for the sake of clarity. The active site metals, A and B, are indicated as gold spheres, a well-ordered water molecule is shown as a red sphere. The primer terminus is indicated with a star. The image was created in Molscript [18] using a ternary complex structure of the RB69 Pol (PDB ID code: 3SNN) [19].

In the work presented here, we attempted to dissect the roles of amino acids located outside the RB69 DNA Pol active site and are not directly involved in DNA or dNTP binding. We analysed the effect on DNA polymerization of alanine substitutions of several residues distant from the active site: E614, N711, D714 and Y720. Residues N711 and D714 are located at the base of a β-hairpin (β-strands 25-26) that establishes contacts with the primer strand, while Y720 seems important to position β-strand 28, which forms a β-sheet together with β-strands 25-26 (Figure S1) [5]. Residue E614, on the other hand, has been proposed to be part of a putative RNA binding motif [15], which seems to also be involved in the alignment of the primer terminus [16]. We found a weakly conserved palm-subdomain residue, D714 (Figure 1A), which, when replaced with alanine, impairs the replicative functions of the enzyme both *in vivo* and *in vitro*. In the 1.8 Å resolution structure of the catalytic complex of RB69 Pol. The OD1 and OD2 atoms of the D714 side chain make hydrogen bonds with the main-chain amide of G717 and the N-ε of R719, and contact the guanidine group of R685 and a well-ordered water molecule [6,19] (Figure 1B). Given the distance of D714 from the catalytic center and the severity of the observed phenotype, we sought to understand the molecular contribution of D714 to the polymerase function.

To that end, we have biochemically characterized the RB69 Pol^D714A^ *in vitro* and solved three X-ray crystallographic structures of the mutant polymerase. Taken together, our data demonstrate how subtle structural changes originating from the substituted D714 can “act at a distance”, and propagate through neighbor-to-neighbor interactions, eventually reaching the catalytic center of the enzyme to distort its geometry.

## Materials and Methods

### Construction, expression and purification of RB69 DNA Pol mutants

Plasmids expressing alanine mutants in E614, N711, D714 and Y720 of RB69 DNA Pol were generous gifts from Jim Karam (Tulane University) and are derivatives of plasmids pCW19R and pCW.50R. Plasmid pCW19R encodes the wild-type RB69 gp43 gene under control of the T7 promoter in the pSP72 vector (Promega). Plasmid pCW.50R expresses a substituted RB69 DNA Pol with D222A and D327A replacements that eliminate the 3′-5′-exonuclease activity of the enzyme. For the D714A RB69 DNA Pol variant, we repeated the construction of the D714A mutation for both expression plasmids using the Quick Site-Directed Mutagenesis protocol (Stratagene). We then reverted the mutated gp43 gene at the 714 site and used these plasmids in *in vivo* DNA synthesis experiments as independent controls to confirm the observed phenotype. Additionally, for crystallographic purposes the D714A substitution was combined with Y567A replacement. The presence or absence of mutations in protein expression vectors was verified by sequencing of the entire open reading frame. Enzymes that are reported in this study and designated Pol^+^Exo^+^, Pol^D714A^Exo^+^ or Pol^+^Exo-, Pol^D714A^Exo-, Pol^Y567A/D714A^Exo- originate from the pCW19R and pCW.50 plasmids, respectively.

Expression and purification of Pol^+^Exo^+^, Pol^+^Exo-, Pol^D714A^Exo^+^, Pol^D714A^Exo- and Pol^Y567A/D714A^Exo- gp43s were performed as previously described [16]. For crystallography experiments requiring milligram quantities of protein, the purification procedure was significantly modified. The plasmid expressing the Pol^D714A^Exo- or Pol^Y567A/D714A^Exo- mutant was introduced into BL21(DE3) CodonPlus RIL cells (Stratagene) and after induction with 0.5 mM IPTG, bacteria were cultured for 18 h at 15°C. Frozen pellets were resuspended in a A_0.5_ buffer containing 50 mM Tris-HCl, pH7.8, 500 mM NaCl, 5% (w/v) glycerol, 1 mM EDTA, pH 8.0, 1 mM DTT, 0.1 mM PMSF in the presence of protease inhibitor cocktail (Roche). Cells were sonicated three times for 30 sec. on ice, centrifuged and the clear lysate was passed through a 5 ml Q column (GE HealthCare) to remove DNA. The flow-through was subsequently diluted with A_0_ buffer (50 mM Tris-HCl, pH 7.8, 5% (w/v) glycerol, 1 mM EDTA, pH 8.0, 1 mM DTT, 0.1 mM PMSF) to reduce the salt concentration to approximately 80 mM, and loaded on the second 5 ml Q column. The protein was eluted with a linear gradient of 75 ml of 0.05 M - 1 M NaCl in A_0_ buffer. Peak fractions were pooled and the buffer was exchanged to B_0_ (50 mM MOPS, pH6.8, 5% (w/v) glycerol, 1 mM EDTA, pH 8.0, 1 mM DTT, 0.1 mM PMSF) with 200 mM NaCl. The sample was then loaded on a 5 ml heparin column (GE HealthCare) and eluted with a linear gradient of 100 ml of 0.2 M – 1 M NaCl in B_0_ buffer. Peak fractions were pooled and diluted with B_0_ buffer to lower the NaCl concentration to 25mM and applied to a 1 ml MonoS column (GE HealthCare). The protein was eluted in a linear gradient of 20 ml of 0.025 M – 0.3 M NaCl in B_0_ buffer. Fractions were concentrated to 18.5 – 20 mg/ml in 10 mM Tris-HCl, pH7.5, 10 mM KCl, 2.5% (w/v) glycerol, 3 mM DTT and flash frozen in liquid nitrogen.

### DNA synthesis *in vivo*

DNA synthesis by different RB69 DNA Pol variants *in vivo* was measured using a T4 phage growth complementation assay and a radioactive thymidine incorporation assay. The experiments were performed as described previously [7,16].

### Forward mutagenesis assay in lacZα gene *in vitro*

Mutation tests *in vitro* on the M13mp2 *lacZα* substrate were performed according to the published protocol [21]. The incubation mixtures (25 µl) contained 150 ng of a gapped M13mp2 *lacZα* DNA substrate, 6 pmol of Pol^D714A^Exo^+^ or 3-10 pmol of Pol^D714A^Exo-, and 250 µM of each dNTP in a polymerase activity buffer (25 mM Tris-acetate (pH7.5), 10 mM Mg-acetate, 150 mM K-acetate, 2 mM DTT). Reactions were incubated for 30 min. at 37°C, stopped by addition of 0.5 M EDTA (pH 8.0) and analyzed on agarose gels. Products of complete gap-filling reactions were introduced into M1061 cells and plated on CSH50 detector strain to score blue M13 plaques (correct synthesis) and light blue or colorless plaques (error-prone synthesis) as described previously [7,20].

### Preparation of DNA substrates

All oligonucleotides used in this study, except for the nucleotides used in crystallization trails, were synthesized in the Laboratory of DNA Sequencing and Oligonucleotide Synthesis (Institute of Biochemistry and Biophysics, Warsaw, Poland) and gel-purified. Primers were 5ʹ-end radiolabeled with [γ-P^32^]-ATP (10 mCi/ml, 3000 Ci/mmol; Hartmann Analytic) by T4 DNA polynucleotide kinase (Takara) and annealed to the corresponding templates at a molar ratio of 1:1.3 in 10 mM Tris-HCl, pH7.5. Annealing reactions were heated at 85°C for 5-10 min. and allowed to cool slowly to 25°C. Samples were kept at 4°C or frozen prior to usage. The oligonucleotides used in crystallization experiments were synthesized using an in-house oligonucleotide synthesizer. The non-hydrolysable nucleotide analog dGpnpp (2ʹ-deoxy-guanosine-5ʹ-(α,β)-imido]triphosphate) was purchased from Jena Bioscience (Gmbh).

### DNA binding and exonuclease activity assays

DNA binding affinities of different gp43s were determined by DNA gel retardation assays as described [16] with slight modifications with regards to the length and the sequence of the primers, as well as enzyme concentration. 50 nM of a 5ʹ-radiolabeled 20-mer primer (5ʹ-AGCTACGCGGCTGTCATAAG-3ʹ) annealed to a 26-mer template (5ʹ-TTGCGTCTTATGACAGCCGCGTAGCT-3ʹ) were incubated with varying amounts of RB69 DNA Pols (7.5 nM – 500 nM for Exo+ and 2.5 nM – 400 nM for Exo- variants, respectively). The fraction of bound DNA was calculated as the ratio of intensity of all shifted species at a given protein concentration divided by the total. The apparent dissociation constant K_*Dapp*_ for DNA was estimated from fitting the shifted DNA fraction (y; nM) versus protein concentration (x; nM) with the equation y = mx/(x +K_*Dapp*_), where m is a scaling factor. Curve fitting was performed with KaleidaGraph 4.03 (Synergy Software).

The exonuclease activity of the Pol^D714A^ was tested on the same DNA substrate as used in DNA binding assay. The reactions were performed as described elsewhere [16].

### Pre-steady state kinetic analysis of correct nucleotide incorporation

Chemical quench experiments were performed at 10°C using a KinTek RQF-3 instrument (KinTek Corp.). Single turnover conditions were achieved by maintaining a 10-fold excess of RB69 DNA Pol over the 5ʹ-radiolabeled 13*/19-mer DNA duplex (primer: 5ʹ-GCGGACTGCTTAT-3ʹ; template: 5ʹ-TCAGTATAAGCAGTCCGCG-3ʹ). Briefly, enzyme and DNA from one syringe were rapidly mixed with MgSO_4_ and various concentration of dNTP from the other syringe. The reaction was quenched with 0.5 M EDTA (pH 8.0) after different times ranging from 0.003 sec - 1.75 sec. The final concentrations after mixing were as follows: 1 µM of enzyme, 100 nM of DNA, 0.001 mM – 1.5 mM dATP and 10 mM MgSO_4_ in a polymerase activity buffer (66 mM Tris-HCl (pH 7.6), 100 mM NaCl). The reactions were repeated 2 - 4 times for each dATP concentration per each enzyme. Products and substrates were separated on 16% denaturing polyacrylamide gels and visualized on a phosphoimaging scanner (Amersham Biosciences). Product formation curves were fitted to a single exponential equation [Product _DNA+1_] = A(1 – e^-kobst^), where A – amplitude, t – time (sec), to obtain k_*obs*_ (Figure S2). The k_*obs*_ values were plotted against dATP concentration and the resulting curve was fit to a hyperbola k_*obs*_ = k_*pol*_ [dNTP]/K_*d,dNTP*_ + [dNTP], where k_*pol*_ represents the rate of phosphoryl transfer and K_*d,dNTP*_ is the dissociation constant of the incoming dNTP. Curve fitting was performed with GraphPad Prism 4 (Life Sciences Co., Stamford, CT).

### Crystallization of RB69 Pol^D714A^ apoenzyme and ternary complex

Crystals of exonuclease deficient RB69 Pol^D714A^ apoenzyme and Pol^Y567A/D714A^ catalytic complex were grown using the vapor diffusion method at room temperature. In order to obtain the apoenzyme crystals, equal volumes (2 µl) of the concentrated polymerase (18.5 mg/ml) and the reservoir solution (1.6 M (NH_4_)_2_SO_4_, 0.01 M MgCl_2_ and 0.05 M sodium dihydrogen citrate, pH 5.6) were mixed and allowed to equilibrate against 0.5 ml of the well solution. The largest crystals (0.3 µm – 0.5 µm) grew within 3 – 5 days, were transferred to the cryoprotectant (1.65 M (NH_4_)_2_SO_4_, 0.01 M MgCl_2_, 0.05 M sodium dihydrogen citrate, pH 5.6, 3 mM DTT, 10 mM KCl, 20% (w/v) glycerol) and subsequently flash frozen in liquid nitrogen. The ternary complex of Pol^Y567A/D714A^ was prepared by mixing the protein (20 mg/ml) with a freshly annealed 13/18-mer DNA duplex (primer: 5′-GCGGACTGCTTAC-3′; template: 5′-TCACGTAAGCAGTCCGCG-3′) in 1:1.2 molar ratio, respectively. DNA annealing buffer contained 10 mM CaCl_2,_ 50 mM NaCl and 20 mM Tris-HCl, pH 7.6. The non-hydrolysable dGpnpp analog was subsequently added to the mixture to the final concentration of 2 mM. Crystals for the Ternary I structure were grown via hanging-drop vapor diffusion by mixing equal volumes of the ternary complex and the reservoir solution containing 10% (w/v) PEG 8,000, 0.2 M (NH_4_)_2_SO_4_ and 0.1 M sodium citrate, pH 5.6. The crystals appeared within two days and were cryoprotected in the reservoir buffer with increased 14% (w/v) PEG 8,000 and 25% (w/v) glycerol, and flash frozen in liquid nitrogen. For Ternary II, crystals were grown using the same method as Ternary I, but with reservoir solution containing 12% (w/v) PEG 8,000, 0.2 M (NH_4_)_2_SO_4_, and 0.1 M MES, pH 6.0. The crystals appeared within two weeks and were cryoprotected in the same manner as Ternary I.

X-ray data collection was performed at beamlines X12C and X25 of the National Synchrotron Light Source at Brookhaven National Laboratory (Uptown, NY). Datasets were processed using HKL2000 [21] (apo structure), or XDS [22] and Scala [23] as implemented in the autoPROC pipeline [24] (ternary structures). The Pol^D714A^ apoenzyme and both Pol^Y567A/D714A^ ternary complex structures were solved by molecular replacement in Molrep [25] using the wild-type RB69 DNA Pol apoenzyme structure (PDB ID code: 1IH7) [3] and Pol^Y567A^ catalytic complex (PDB ID code: 3NGI) [13] as starting models, respectively. The structures were refined using Phenix [26], followed by manual model building in Coot [27]. Validation of structures was performed with MolProbity [28]. Coordinates for the apoenzyme and both ternary complex structures were deposited in the Protein Data Bank with accession codes 4I9L, 4I9Q, and 4KHN, respectively. The crystals of the apoenzyme contained one molecule in the asymmetric unit (ASU) and the electron density was of sufficient quality to build the entire polypeptide chain (residues 1 - 903). The structure contained one GMP molecule located far from the catalytic center, as observed for the wild type apoenzyme structure [3]. The Ternary I structure contained two molecules in the ASU with overall nearly identical conformations (RMSD of 0.65 Å for 831 C-α atoms). The electron density was of sufficient quality to build most of the protein (residues 1-895 in molecule A and 1-900 in molecule B), DNA and the incoming triphosphate. In both molecules in the ASU the fingers subdomain was partially disordered (residues 497-545 in molecule A and 504-530 in molecule B). Each protein molecule contained an extra dGpnpp molecule. The Ternary II structure also contained two molecules in the ASU with nearly identical conformations (RMSD of 0.4 Å for 759 C-α atoms). The electron density was of sufficient quality to build most of the protein (residues 1-901 in both molecules), DNA, and the incoming triphosphate. In molecule B the fingers subdomain was partially disordered (residues 504-534). Each protein molecule contained an extra dGpnpp molecule.

## Results

### D714 is essential for phage growth

The importance of the E614, N711, D714 and Y720 side chains was examined by studying the impact of their alanine substitutions on DNA polymerization. The phenotypes of the point mutants were verified using an *in vivo* T4 phage growth complementation assay that is mainly dependent on the catalytic activity of the mutant polymerase. We assessed the essentiality of E614, N711, D714 and Y720, for *in vivo* DNA synthesis by RB69 Pol using a combination of T4 phage growth complementation assay and radioactive thymidine uptake experiments, as described previously [7]. Both tests utilize the unique feature of T4 and RB69 DNA Pols to substitute for each other during phage DNA replication *in vivo*. Briefly, the T4 phage *43amam* mutant, whose endogenous DNA polymerase is inactivated by two amber mutations at the 202 and 386 codons of the T4 gp43 gene, is used to infect the *E. coli* BB strain. *E. coli* BB cells carry a plasmid expressing either wild type or mutant RB69 Pol, which in the absence of T4 Pol is the only enzyme capable of performing phage genome DNA replication [7,16]. Both phage growth and DNA synthesis levels were monitored. The rate of radioactive thymidine incorporation to T4 *43amam* phage genomic DNA was measured after the host DNA synthesis had ceased completely [7]. Phage growth appeared to be normal for the E614A, N711A and Y720A mutants (Table 1). However, phage growth for Pol^D714A^ was severely inhibited in this assay, suggesting that D714 is essential for replication *in vivo* (Table 1). In order to confirm the importance of D714, we introduced additional substitutions (D222A and D327A), which render the mutant polymerase deficient for exonucleolysis. Similarly, the D714AExo- mutant was unable to fully support phage growth. Both variants displayed reduced DNA synthesis levels *in vivo*, further supporting the notion that the D714A replacement affects DNA polymerization. As shown in Table 1, radioactive thymidine uptake was decreased to 10% in cells expressing both the Pol^D714A^Exo^+^ and Exo- mutants compared to those expressing wild type RB69 DNA Pol. However, the growth of wild type T4 *43* ^*+*^ phage was unaffected, indicating that the RB69 *43* D714A mutation is recessive.

**Table 1:** Relative DNA synthesis *in vivo*.

Polymerase	Growth on T4 *gp43am*	Growth on T4 *gp43wt*	Relative DNA synthesis
Pol^+^Exo^+^	+	+	1.0
Pol^+^Exo-	+	-	0.9
Pol^D714A^Exo^+^	-	+	0.1
Pol^D714A^Exo-	-	+	0.1
Pol^E614A^Exo^+^	+	+	ND
Pol^N711A^Exo^+^	+	+	ND
Pol^Y720A^Exo^+^	+	+	ND

Phage growth was estimated in the complementation assay as described in detail elsewhere [7]. Relative DNA synthesis was normalized to [^3^H]-thymidine incorporation 22 - 37 min. after infection with T4 *43am*. The value 1.0 for Pol^+^Exo^+^ represents 1.5 × 10^5^ dpm ^3^H per 10^7^ infected cells. The relative DNA synthesis levels were not determined (ND) for E614A, N711A and Y720A mutants as they supported the growth of the T4 *gp43am* phage *in vivo*.

### Fidelity of RB69 Pol^D714A^ mutant

One possible reason for the reduced ability of Pol^D714A^ to carry out DNA replication *in vivo* could be impaired nucleotide selectivity. We therefore decided to analyze the fidelity of synthesis of the D714A mutant using a genetic forward mutation assay [20]. Purified Pol^D714A^Exo^+^ or Pol^D714A^Exo- were used to perform synthesis of a *lacZ* template sequence in a gapped M13mp2 DNA substrate *in vitro* (Table 2). D714A gp43 was able to fill the 407 nt gap, and resulted in a *lacZα* mutator frequency (5 x 10^-4^) that is almost indistinguishable from the Pol^+^Exo^+^ (8 x 10^-4^), and is similar to background values for uncopied M13mp2 DNA (5 – 7 x 10^-4^) [20,29]. Moreover, the Pol^D714A^Exo- variant displays a mild but reproducible 4.8-fold antimutator effect compared to the wild type Exo- polymerase (6 x 10^-4^ versus 29 x 10^-4^, respectively), suggesting that, in fact, the D714A substitution results in an enzyme with higher fidelity than the wild type polymerase.

**Table 2:** Fidelity of Pol^D714A^ in a *lacZα* forward mutagenesis assay *in vitro*.

Polymerase	Total number of plaques	Mutant plaques	Correction factor	*lacZα*mutation frequency x 10^-4^
Pol^+^Exo^+^	54 411	52	0.88	8
Pol^+^Exo-	91 799	276	0.95	29
Pol^D714A^Exo^+^	26 009	13	1	5
Pol^D714A^Exo-	74 040	62	0.69	6

The entries for Pol^+^Exo^+^ and Pol^+^Exo- were adapted from a previous report [7]. Mutants from Pol^D714A^Exo^+^ were not sequenced, so the correction factor value was assumed to be 1.

### DNA binding affinity and exonuclease activity

**Figure 2:**
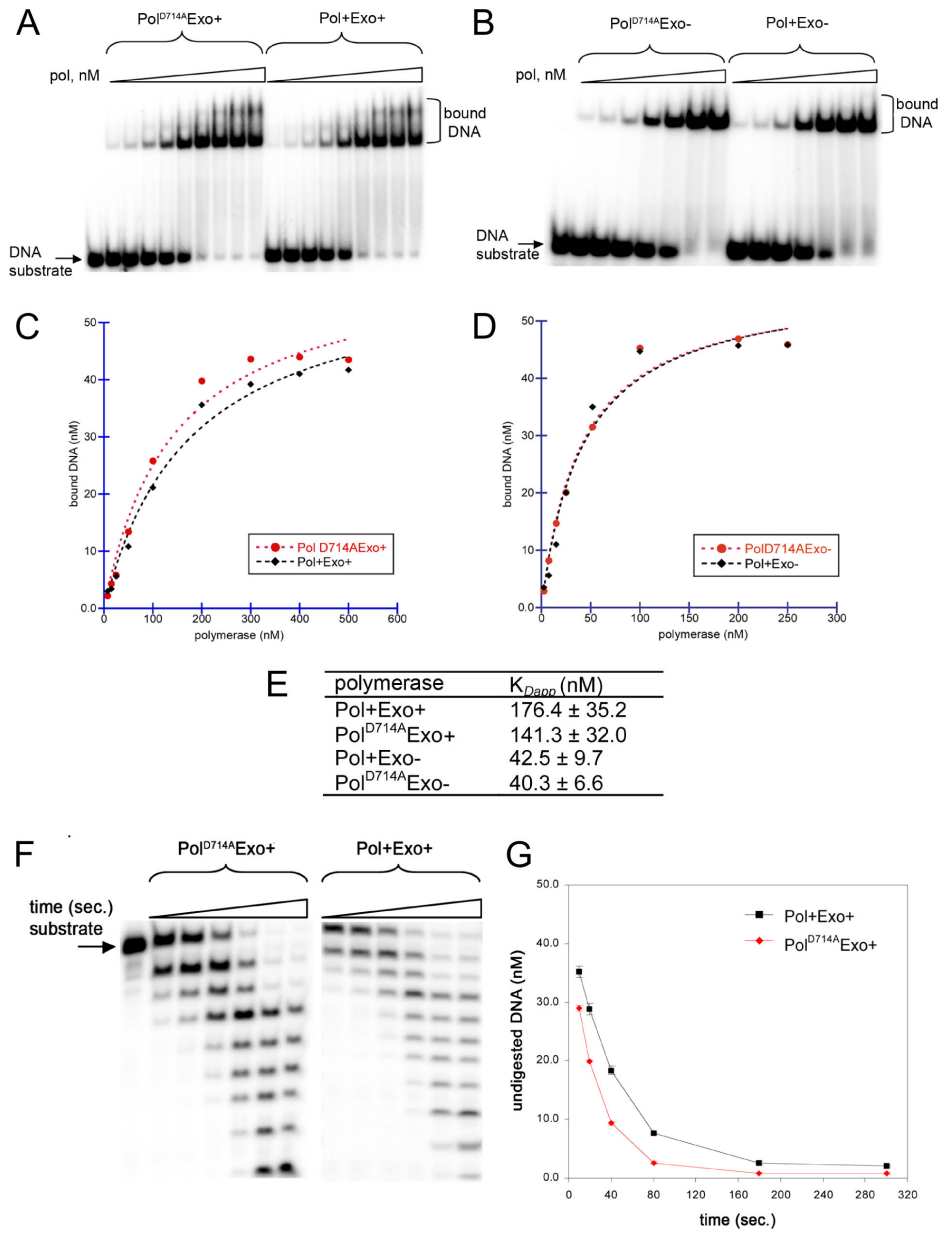
DNA binding affinity and exonuclease activity of Pol^D714A^ on dsDNA. DNA binding affinities of the wild type RB69 and Pol^D714A^ polymerases (**A**), as well as their exonuclease deficient derivatives (**B**), were determined by DNA mobility-shift assays. A radiolabeled 20*/26-mer primer-template DNA substrate was incubated with increasing amounts of each polymerase, and the resulting DNA-protein complexes were analyzed on native 6% polyacrylamide gels (**A** and **B**). The amount of bound DNA substrate was quantified as an average from two independent experiments and plotted against protein concentration (**C** and **D**). (**E**) K_*Dapp*_ was calculated using Kaleidagraph (Synergy Software). The additional shifted DNA species visible for Exo^+^ variants at higher protein concentrations may correspond to more than one molecule of polymerase bound per oligonucleotide substrate. (**F**) A ^32^P-labeled 20*/26-mer DNA substrate was incubated with the mutant or wild-type RB69 DNA Pol for 10, 20, 40, 80, 180 and 300 sec. at 37°C. Products of DNA degradation were analyzed on denaturing polyacrylamide gels and visualized on a phosphorimager. (**G**) The amount of undigested DNA substrate was calculated and plotted as a function of time. Data are averages from three independent experiments for each polymerase. Pol^D714A^Exo^+^ displays slightly elevated exonuclease activity, reflected in an only ~ 10% increase in DNA substrate consumption at the shortest incubation times. The observed difference between the exonucleolytic activities of the enzymes appears therefore to be negligible.

The observed antimutator phenotype of D714A polymerase *in vitro* may result from increased exonuclease activity and/or decreased ability to form binary polymerase-DNA complexes [11,16]. Thus, we decided to test whether the D714A substitution might decrease the DNA binding affinity of the polymerase and/or change its ability to exist in the editing mode required for efficient proofreading. We characterized the relative dsDNA binding abilities of the mutant and wild type enzymes in both exonuclease backgrounds using DNA gel retardation assays (Figure 2A and B). Binding of either polymerase to a radiolabelled 20*/26-mer DNA substrate increased proportionally with the enzyme concentration. The K_*Dapp*_ was calculated as described in Materials and Methods (Table 2, Figure 2C, D and E). The wild type RB69 DNA Pol displays weaker dsDNA binding than its Exo- derivative, which is consistent with recently published data [13] and comparable to what was observed for T4 polymerase ( [15,30] and reference therein) (Figure 2E). However, the Pol^D714A^Exo^+^ and Pol^D714A^Exo- bind dsDNA with almost the same affinity as the wild-type RB69 Pol and its exonuclease deficient derivative, respectively. Slightly higher affinity of the Pol^D714A^Exo^+^ comparing to the Pol^+^Exo^+^ (K_*Dapp*_ of 141.3 nM versus 176.4 nM, respectively) remains within the experimental error range (Figure 2E).

Furthermore, the D714A replacement does not seem to affect the exonuclease activity of the enzyme *in vitro*, as the mutant hydrolyses dsDNA with similar proficiency as the wild type polymerase (Figure 2F, G). Thus, it appears that the affinity of the mutant for the DNA substrate, as well as its ability to degrade it remains unchanged and cannot explain the observed severe *in vivo* defects.

### Pre-steady state kinetics of correct nucleotide insertion

Finally, we hypothesized that the observed effects might be a consequence of a decrease in polymerization activity. Since steps other than catalysis will affect steady-state rates, we decided to perform single turnover experiments to determine the rate of polymerization for correct nucleotide incorporation. We conducted the reactions at 10°C temperature using a 10:1 ratio of enzyme to DNA substrate. Under such enzyme-saturating conditions presumably all DNA molecules remain bound to the polymerase and enzyme recycling after dNTP incorporation is prevented (no unbound DNA is present in the reaction mixture). The errors introduced by the pre- and post-chemistry steps (i.e. DNA association or release from the polymerase) are therefore eliminated from the calculated k_*pol*_ values [31].

**Figure 3:**
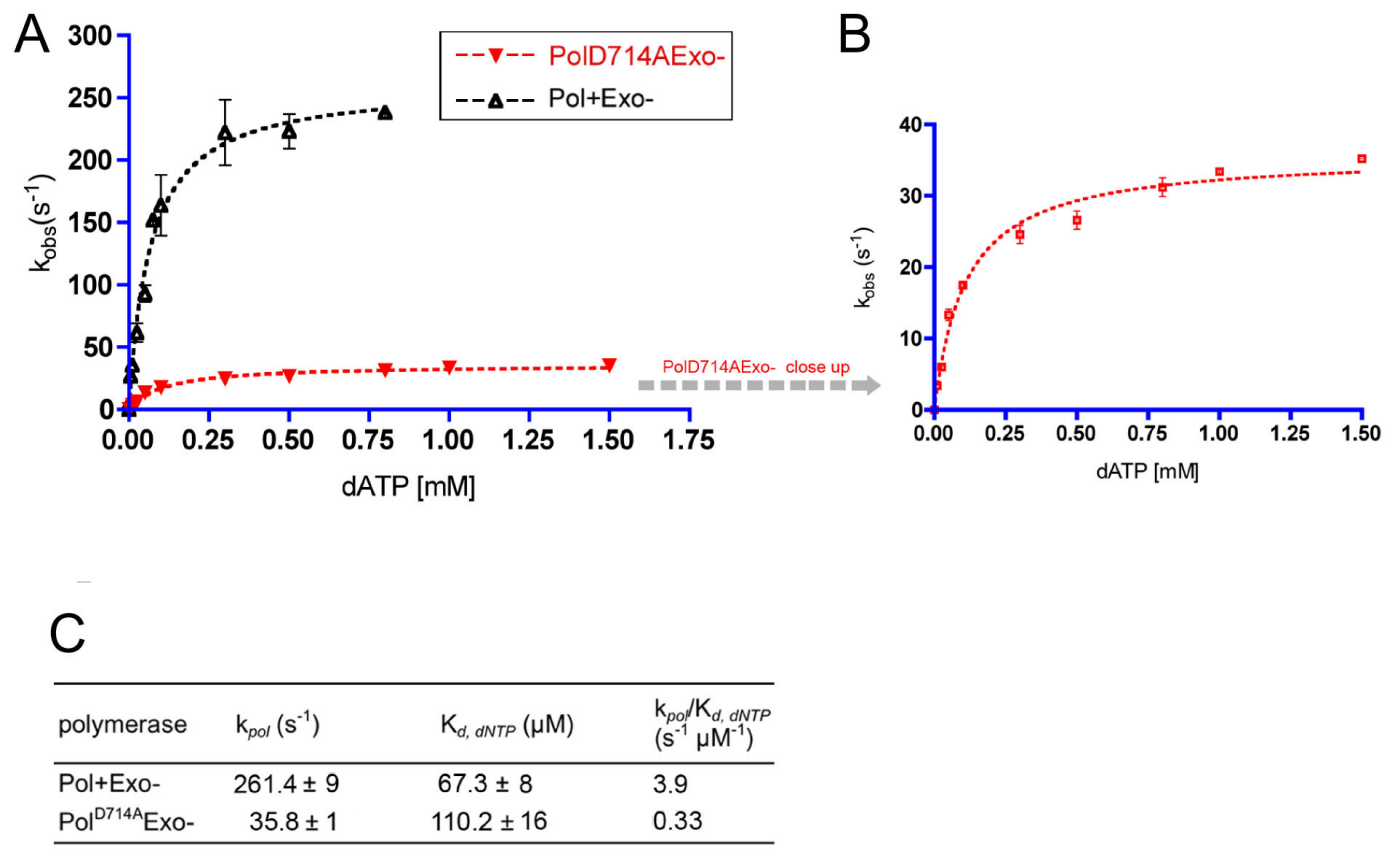
Pre-steady state kinetics of correct nucleotide incorporation by the Pol^D714A^Exo- mutant. Reactions to measure the incorporation of dATP opposite template T by RB69 DNA Pols were carried out at 10°C. A pre-incubated solution containing the enzyme (1 µM) and radiolabeled 13*/19-mer DNA substrate (100 nM) was mixed with 10mM MgSO_4_ and dATP (0.001 mM – 1.5 mM). The reactions were quenched by addition of 0.5 M EDTA (pH 8.0) and analyzed on denaturing polyacrylamide gels. The data were fit to a single-exponential equation to obtain k_*obs*_ (Figure S2). These values were subsequently plotted as a function of dATP concentration for D714A and wild type RB69 DNA Pols and k_*pol*_ and K_*d,dNTP*_ were calculated as described in Materials and Methods. The standard deviations (SD) are shown as error bars (**A**) and ± values (**B**). A close-up of the mutant plot is depicted on the right. The catalytic efficiency of each polymerase was obtained by dividing its respective k_*pol*_ by K_*d,dNTP*_ (**B**).

The kinetic parameters k_*pol*_ and K_*d,dNTP*_ measured for the wild type RB69 Pol in this study remain similar to the values observed by others for correct base pair formation [9,12,13,32,33] (Figure 3 and Figure S2). Pol^D714A^ exhibited only a 2-fold higher dissociation constant (*K*_*d,dNTP*_) for the complementary dATP compared to the wild type RB69 Pol (110.2 µM versus 67.3 µM, respectively) (Figure 3B). However, the maximum rate of DNA polymerization (*k*_*pol*_) of Pol^D714A^ was reduced by 8-fold, resulting in a considerable 12-fold decrease in catalytic efficiency for proper Watson-Crick base pairing (Figure 3). This substantial drop in the polymerization activity provides a concrete explanation for the detrimental effects of D714A replacement observed *in vivo.*

### Crystal structure of the Pol^D714A^ apoenzyme

**Figure 4:**
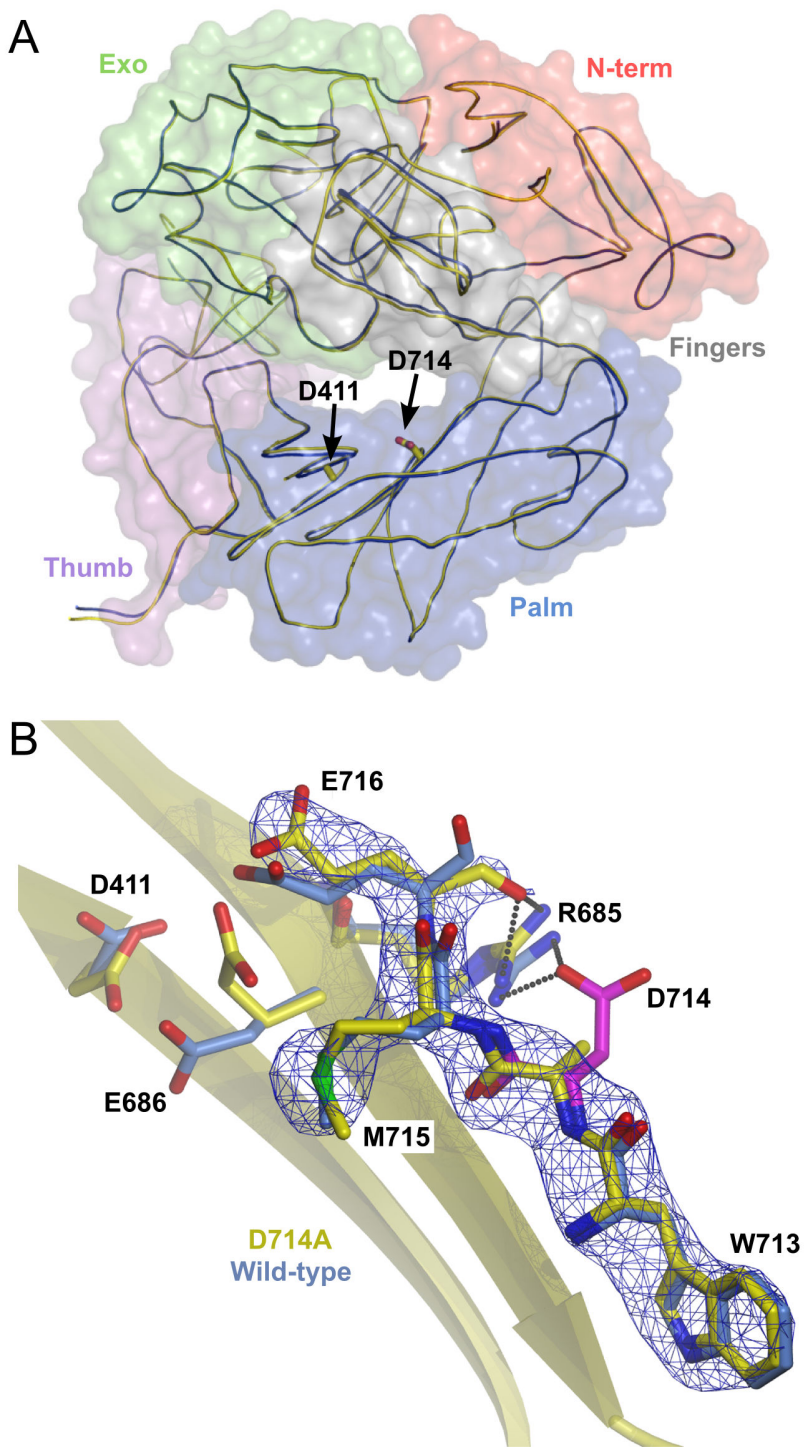
Crystal structure of the Pol^D714A^ mutant at 2.60 Å resolution. (**A**) Overlay of the wild type (PDB ID code 1IH7; navy blue) and Pol^D714A^ apoenzyme structures (PDB ID code: 4I9L; yellow), showing the overall fold of the mutant enzyme. The positions of the D714 and D411 residues of the wild type RB69 Pol are indicated. Polymerase subdomains are shown as transparent surfaces in different colors. (**B**) Simulated annealing omit map contoured at 1.5σ, showing the region surrounding D714 in the Pol^D714A^ crystal structure (yellow), overlaid with the corresponding region of the wild type RB69 Pol structure (cyan). D714 of RB69 Pol is colored magenta; hydrogen bonds between interacting residues are shown as dashed lines.

To gain insight into the origin of the unexpected decrease in polymerization rate of the D714A mutant we decided to solve the crystal structure of the exonuclease deficient Pol^D714A^ apoenzyme (PDB ID code: 4I9L, Table 3). Pol^D714A^ is structurally very similar to the 2.21 Å wild type RB69 DNA Pol apoenzyme (PDB ID code: 1IH7), with an RMSD value of 0.4 Å for the backbone atoms (Figure 4A). This low RMSD value indicates that the overall fold of the mutant polymerase remains unchanged. Inspection of the omit map calculated for the 712-724 residues of Pol^D714A^ structure, at a contour level as low as 1.5σ, confirms the presence of the D714A substitution, as well as the appearance of structural distortions in the vicinity of the replaced residue (Figure 4B). These changes are most probably the immediate consequence of the abrogation of polar D714 side chain interactions. This includes an E716-G717 peptide bond flip, resulting in formation of a salt bridge between the main chain carbonyl oxygen of E716 and the guanidine group of R685, which in the wild type enzyme contacts the D714 side chain (Figure 4B). The new conformation of E716 appears to be stabilized by a 2.7 Å hydrogen bond between the O-ε atoms of E716 and E686. This interaction is most likely facilitated by a change in the local chemical microenvironment, maintaining one of the carboxylic acid groups in a protonated state in order to avoid electrostatic repulsion [34].

**Table 3:** X-ray data collection and refinement statistics for the Pol^D714A^ crystal structures.

		**Apoenzyme**	**Ternary I**	**Ternary II**
**Data collection statistics**				
Space group		*P 2_1_ 2_1_ 2_1_*	*P 1 2_1_ 1*	*P 1 2_1_ 1*
Cell dimensions				
	*a*, *b*, *c* (Å)	116.88,199.37,80.22	73.78,119.41,146.02	74.29,119.40,148.10
	α, β, γ (°)	90, 90, 90	90.00, 90.26, 90.00	90.06, 91.64, 90.02
Wavelength (Å)		1.0	1.1	1.1
Resolution (Å)		33.24−2.58 (2.66−2.58)	38.40−2.30 (2.31−2.30)	35.33−2.55 (2.56−2.55)
R_merge_		0.14 (0.829)	0.075 (0.331)	0.063 (0.617)
I / σI		15.2 (2.5)	12.6 (3.5)	16.8 (2.5)
Completeness (%)		99.5 (100)	98.6 (99.4)	99.9 (99.8)
Multiplicity		6.2 (6.3)	3.2 (3.4)	4.9 (4.8)
**Refinement Statistics**				
Resolution (Å)		33.24−2.58	38.40−2.30	35.33−2.55
Unique reflections		52,147	110,586	83,995
*R*_work_ / *R*_free_		0.2145/0.2489	0.2503/0.2877	0.1682/0.2109
No. atoms				
	Total	7,642	15,972	15,657
	Protein	7,384	14,183	13,928
	DNA	–	1,256	1,256
	Ligands/Ions	38	128	169
	Water	220	406	304
*B*-factors				
	Protein	62.8	36.5	65.3
	DNA	–	52.3	72.6
	Ligands/Ions	82.7	50.8	86.0
	Water	58.7	32.9	50.0
RMS deviations				
	Bond lengths (Å)	0.008	0.014	0.011
	Bond angles (°) Ramachandran	1.016	1.035	1.192
	Favored (%)	95.34	96.3	96.3
	Outliers (%)	0.33	0.40	0.30
**PDB ID**		4I9L	4I9Q	4KHN

The interaction between E716 and E686 is not seen in the wild type RB69 gp43 apoenzyme structure, and might favor the conformations observed in the mutant structure. The movement of E716 most likely triggers a shift of its surrounding residues, in particular R719, Y720, E722 and K724, which comprise the 27-28 β-sheets surface of the RB69 Pol palm subdomain. The electron density for R719 is wider, suggesting that it may dynamically adopt two conformations. Importantly, the distortions of the hydrogen bond network around the A714 amino acid domino out towards the polymerizing center of the enzyme and eventually affect the position of the catalytically essential D411. The defined orientation of this aspartate is critical for the optimal geometry of the metal ion-binding site during nucleotydyl transfer [5,6]. The D411 residue, located approximately 15 Å away from D714, displays a large rotation of the side chain and a slight 0.3 Å shift of the C-α atom (Figure 4B). These rearrangements are most likely a consequence of the new conformation of E686, as both residues are spatially close in the native apoenzyme structure.

### Crystal structure of a Pol^Y567A/D714A^ ternary complex

It is possible that the long-distance perturbations observed in the Pol^D714A^ apoenzyme structure would alter the active site conformation and therefore influence the rate of DNA polymerization. To address this possibility we aimed to crystallize the Pol^D714A^ enzyme in the presence of DNA and the incoming nucleotide. Unfortunately, the crystals diffracted poorly, impeding reliable structural analysis. Therefore, we took advantage of the Y567A substitution that has been shown to improve crystal quality without affecting the catalytic parameters of the enzyme ( [13,19,32] and references therein). We obtained crystals of a complex of the mutant polymerase, DNA and the dGpnpp non-hydrolyzable analog (see Materials and Methods) that diffracted to 2.30 Å resolution (ternary complex I). The structure was solved by molecular replacement using Pol^Y567A^ ternary complex structure (PDB ID code: 3NGI) [13] as a search model and resulted in interpretable electron density for most of the protein, DNA and the incoming nucleotide (see Material and Methods and Table 3). A subsequent dataset for a crystal originating from slightly different crystallization conditions was obtained to 2.55 Å resolution (ternary complex II; see Table 3). Upon solving the structure, inspection of the active site revealed a different conformation from that observed in the initial crystal (see below). We then collected several additional datasets. In all cases the observed active site conformation was identical to one of the two crystals described below.

#### Ternary complex I

**Figure 5:**
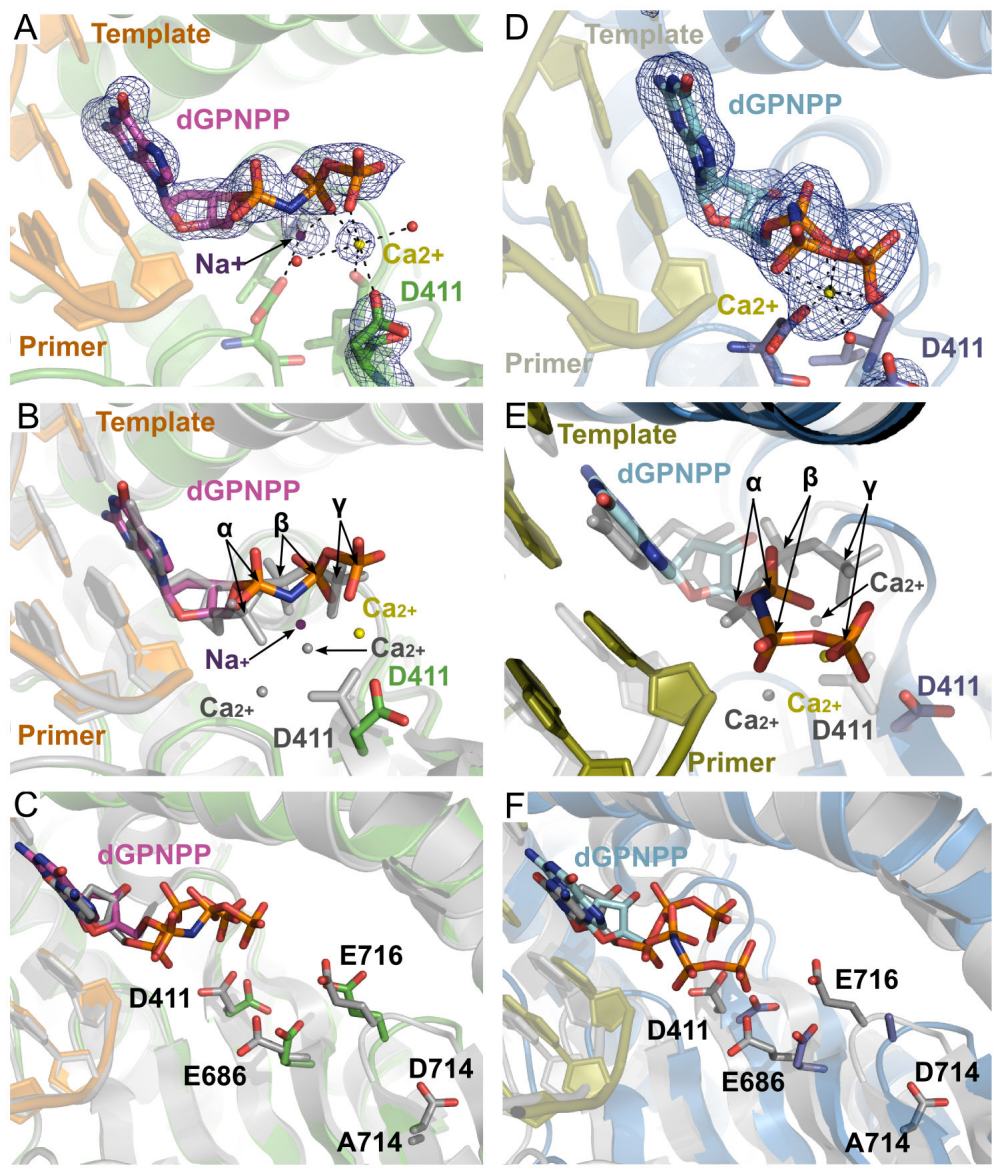
Crystal structures of a catalytic complex of the Pol^Y567A/D714A^ mutant. (**A**) The active site of the mutant in ternary complex I. The end of the primer is shown with incoming dGpnpp (magenta), and bound metal ions: sodium (purple sphere), calcium (yellow sphere), and coordinating waters (red spheres). A simulated-annealing *F*_o_-*F*_c_ omit map, contoured at 3σ, is shown in blue. (**B**) Overlay of ternary complex I (PDB ID code: 4I9Q) and the wild type (PDB ID code: 3NCI) polymerase active sites in their respective ternary complexes highlighting the different conformation of the phosphates of the incoming nucleotide, the metal ions and the flip of the 411 side chain. The D714 mutant structure is rendered with the same colors as in (**A**). The wild type structure is rendered in gray. The α-, β-, and γ-phosphates of the incoming nucleotide are marked with arrows. **C**) A network of interactions propagates the perturbation resulting from the absence of the 714 side chain into the active site, influencing the conformation of D411. The color scheme is as in (**B**). (**D**) The active site in ternary complex II. The DNA (yellow), incoming dGpnpp (cyan) and a calcium ion (yellow sphere) are shown. A simulated-annealing *F*_o_-*F*_c_ omit map, contoured at 4σ, is shown in blue. (**E**) Overlay of ternary complex II (PDB ID code: 4KHN) and the wild type (PDB ID code: 3NCI) polymerase active sites, highlighting the large differences in the conformation of the β- and γ-phosphates of the incoming nucleotide. Like for ternary complex I, a flip in the D411 side chain can be observed relative to the wild-type structure. The α-, β-, and γ-phosphates of the incoming nucleotide are marked with arrows. (**F**) The absence of the 714 side chain in ternary complex II results in similar structural perturbations to those observed in ternary complex I (see C). Due to the lack of the electron density the side chain of E716 was not shown.

As expected, the ternary complex structure reveals an assembled active site with dGpnpp occupying the incoming dNTP binding pocket. The conformation of the template and primer strand is essentially identical to that observed in other RB69 Pol ternary complexes (Figure 5A). Strikingly, however, while the base and sugar of the incoming nucleotide are found in positions that are consistent with other RB69 polymerase structures, both molecules in the ASU present an altered conformation for the three phosphate groups (Figure 5B). As a consequence the α-phosphate is not properly aligned for catalysis. Furthermore, the D714A substitution drastically affects the metal coordination. Neither the dNTP binding metal, nor the catalytic metal ions are found in their expected positions (Figure 5B). Interestingly, this aberrant active site conformation correlates with similar changes to those observed in the apo crystal structure, suggesting that the altered position of the incoming nucleotide is related to the conformation of D411 and, in turn, to the missing D714 side chain. As seen in the apo mutant structure, the D411 is flipped away from the active site and this correlates with the repositioning of the E686 and E716 side chains (Figure 5C, Figure S3B). Thus, the absence of the D714 side chain seems to cause the movement of the key metal-ligating D411 residue in the polymerase pocket and, as such, agrees with the observed decrease in the DNA polymerization rate.

#### Ternary complex II

The overall conformation of the protein is identical to that seen in ternary complex I, and identical to that observed in other RB69 Pol ternary complexes (Figure 5D). In this structure, the phosphate backbone of the incoming nucleotide is severely distorted with respect to the conformation observed in structures of the wt enzyme, and a single divalent (Ca^2+^) metal ion is observed coordinating the β and γ phosphates (Figure 5E). A similar flip in the D411 side chain is observed in this structure, further supporting the idea that the structural perturbations observed in the apo crystal structure contribute to destabilize the active site and result in non-catalytic conformations of the incoming nucleotide (Figure 5F and Figure S3C).

It is important to note that both our ternary complex structures were obtained in a different crystal form than the reference structure (PDB ID code: 3NGI), resulting in a different crystal-packing environment. However, superimposition of both ternary complexes with the wild type structure reveals an identical fold, implying that the observed conformations are not affected by crystal contacts (Figure S4).

## Discussion

We examined the contribution of residues distant to the polymerase active site to the activity of the replicative RB69 DNA Pol. Among the amino acids tested, only the D714A substitution led to the inhibition of T4 *43amam* phage growth (Table 1), implying its impact on catalysis, and we therefore focused on understanding the role of this residue in polymerase activity.

Close analysis of the location of D714 in a 1.8 Å ternary structure of the wild-type RB69 Pol revealed that this residue does not contact DNA or the incoming dNTP [6]. Although the D714 side chain is involved in establishing a hydrogen bond network between G717, R719 and R685 and a well-defined water molecule (Figure 1B), its functions during catalysis remained elusive. The unexpected contrast between the severity of the *in vivo* phenotype and the distance of the D714 residue from the active site posed an intriguing mechanistic question.

We decided to investigate the functional consequences of the D714A substitution that might explain its phenotype. The D714A replacement does not change polymerase fidelity in a forward mutagenesis assay *in vitro* in an Exo^+^ background, whereas the Exo- derivative displayed an approximately 5-fold antimutator effect (Table 2). This is, however, not a consequence of increased DNA dissociation rates or an impaired exonuclease function, supporting the idea that the phenotype of Pol^D714A^ merely results from an altered polymerization activity. As suspected, under single-hit conditions Pol^D714A^ shows a significant 12-fold decrease in the catalytic efficiency implying that the failure of Pol^D714A^ to support growth *in vivo* occurs because of a compromised rate of DNA polymerization. Bearing in mind that direct extrapolation of *in vitro* results to DNA replication *in vivo* is difficult and requires caution, we speculate that the rate of DNA synthesis by Pol^D714A^ *in vivo* is too low to support phage replication, which requires the full duplication of the entire 172-kb T4 genome within 15 min. [35]. A highly efficient polymerase is therefore critical for effective production of phage progeny [36].

We found the drastic effects on DNA polymerization induced by a substitution located far away from the active site to be compelling. In order to understand the mechanism underlying this effect at the atomic level, we first crystallized and solved the structure of the Pol^D714A^ apoenzyme at 2.60 Å resolution. Detailed analysis of the region surrounding the D714 residue reveals, however, an interesting rearrangement of a hydrogen bond network propagating from the site of the substitution towards the catalytic center of the polymerase (Figure 4B). The consequences of these rearrangements are reflected in the new geometry of catalytic D411. The side chain of this catalytic aspartate in its new conformation is flipped away from the active site and points towards E686. In the high resolution crystal structure of RB69 DNA Pol the D411 residue directly coordinates metal ions, whereas E686 and E716 are involved in the formation of a water-mediated hydrogen bond network between D411, the incoming nucleotide and the catalytic metal ions [6,37]. Substitution of these residues to alanine results either in a severe (E686A) or complete (D411A) loss of polymerization activity with a minute impact on dNTP binding [6,37]. Similar effects are observed for the replacement of the corresponding residue to E686 in the homologous yeast Pol δ [2]. However, while the polar character of the D714 side chain appears to be preserved in some B-family enzymes (Figure 1A) and is conserved among T4 phage-like subclass of polymerases (data not shown), this residue in general shows variability. Other amino acids comprising the loop, which emerged to be critical for the interactions, do not display high conservation even in the T4-like polymerases, making it unlikely that the specifics of the mechanism described above apply to other polymerases.

Consistently, ternary complex structures of the D714A mutant reveal that the network of structural perturbations observed in the apoenzyme structure is most likely maintained in the closed conformation of the enzyme, resulting in aberrant metal ion coordination and positioning of the triphosphate of the incoming nucleotide (Figure 5). It is important to mention that in order to obtain a high-resolution crystal structure of the ternary complex it was necessary to introduce an Y567A substitution. While it cannot be completely ruled out that this substitution influences the observed conformation, this possibility seems unlikely given that: (1) the structural perturbations are consistent with those observed in the apoenzyme structure, and (2) the ternary complex of the Y567A mutant (PDB ID code: 3NGI) is structurally indistinguishable from the wild type polymerase. The observed alternation of in the active site geometry propagate towards the catalytic center of RB69 DNA Pol and affect the metal binding site and dNTP alignment, which might conceivably explain the reduced rate of catalysis. An analogous mechanism was proposed for the substitution of the hinge region of the low fidelity β DNA polymerase (Pol β) from X family [38–41]. In Pol β, hinge residues form a hydrophobic patch at the interface between the catalytic and C-terminal domain [41]. These residues are not part of the active center of Pol β. However, their substitution has pronounced effects on Pol β function *in vitro*, resulting in a decreased polymerization activity, impaired nucleotide selectivity and enhanced ability to extend mismatched termini [39].

In conclusion, we propose that a single remote D714 residue indirectly plays a key structural role in the proper alignment of the protein matrix involved in the stability of the metal site in the active site of RB69 Pol. We present biochemical and structural evidence suggesting that a substitution of this residue could alter the hydrogen bond network of its immediate neighbors, spreading the structural perturbation towards the catalytic center of the enzyme and eventually affecting the conformation of residues essential for catalysis. These results should also be considered in light of conformational changes that occur throughout the catalytic cycle of the polymerase [5]. It would be interesting to understand how these perturbations affect the dynamics of the enzyme. Our work can serve as a good example of how residues located distant from the catalytic center can drastically influence polymerase activity [38–48].

## Supporting Information

Figure S1 **Rationale for the mutagenesis.** Residues E614, N711, D714 and Y720 are located in the palm subdomain of the polymerase. N711, D714 and Y720 are part of a β- sheet (show in violet) that contacts the bound DNA. Residue E614 belongs to a putative RNA binding motif (dark purple).
(TIFF)

Figure S2 **Pre-steady state kinetics of the correct nucleotide incorporation by Pol^D714A^ mutant in comparison to the wild type polymerase.** Representative gels and the progress curves for four different dATP concentrations (10 µM, 50 µM, 300 µM and 500 µM) are shown. The goodness of the curve fit (R^2^) and the calculated observed rate constants (k_*obs*_) are included for each nucleotide concentration.
(TIFF)

Figure S3 **Microenvironment near the A714 residue in wild type and ternary complexes I and II.** Stereo images showing the details of the network surrounding residue 714 in the wild type (**A**), ternary complex I (B) and ternary complex II (C). The distances between D411 and E686 are indicated as an arrow (wild type) and dashed lines (ternary complexes I and II, where they form hydrogen bonds).
(TIFF)

Figure S4 **Overlay of the wild type ternary structure with ternary complexes I and II.** C-α traces of the wild type (3NCI, violet), ternary complex I (cyan) and ternary complex II (green). The overall fold of the protein is identical in our structures and the wild type structure with RMSD values of 0.6 Å for 763 C-alpha atoms and 0.5 Å for 815 C-alpha atoms for ternary complexes I and II, respectively.
(TIFF)
