# Supplementary figures and images for: A Remote Palm Domain Residue of RB69 DNA Polymerase Is Critical for Enzyme Activity and Influences the Conformation of the Active Site
Source: PLoS One. 2013 Oct 7;8(10):e76700. doi: 10.1371/journal.pone.0076700 (PMC3792054; doi:10.1371/journal.pone.0076700)

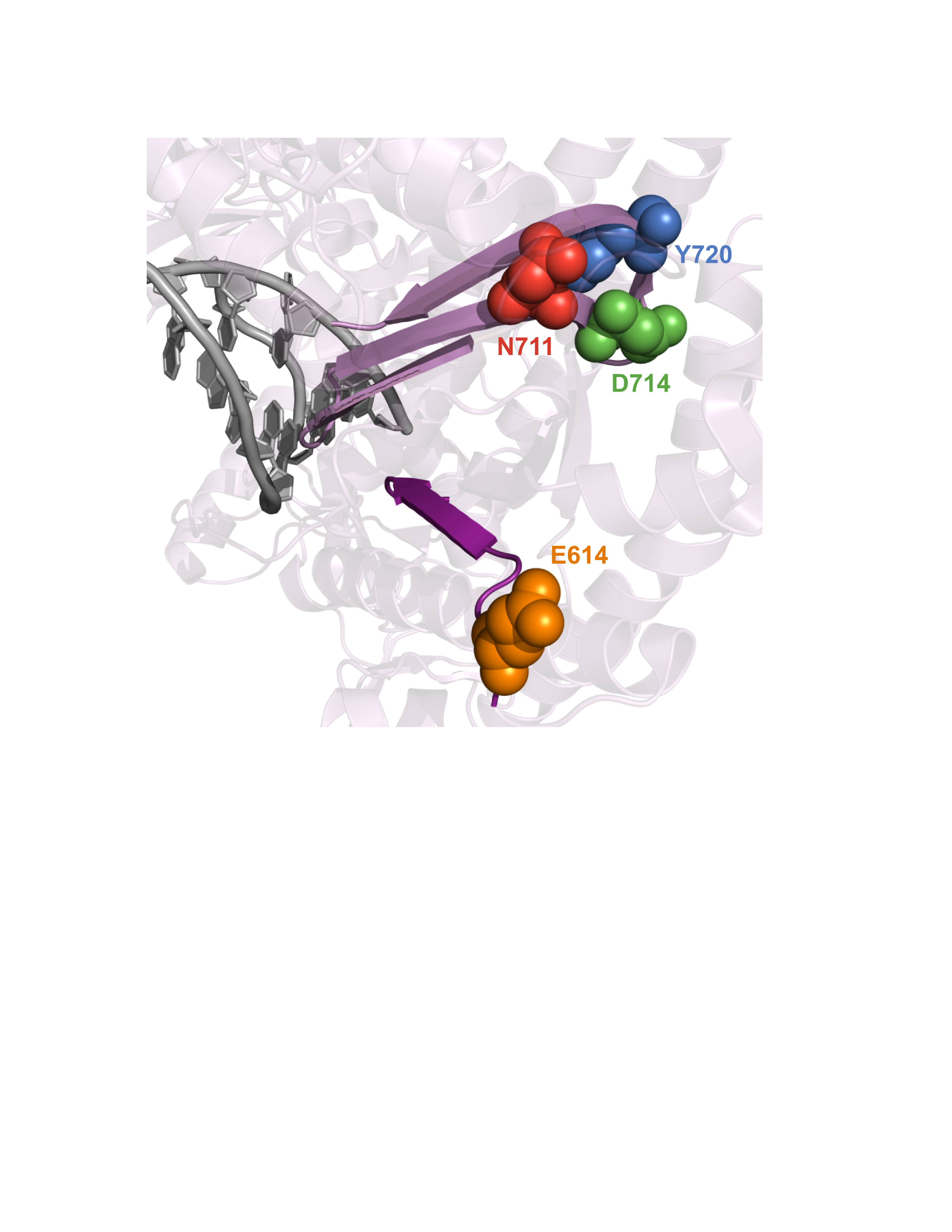

Supplement: Figure S1 — Rationale for the mutagenesis. Residues E614, N711, D714 and Y720 are located in the palm subdomain of the polymerase. N711, D714 and Y720 are part of a β- sheet (show in violet) that contacts the bound DNA. Residue E614 belongs to a putative RNA binding motif (dark purple). (TIFF) [file pone.0076700.s001.tiff]

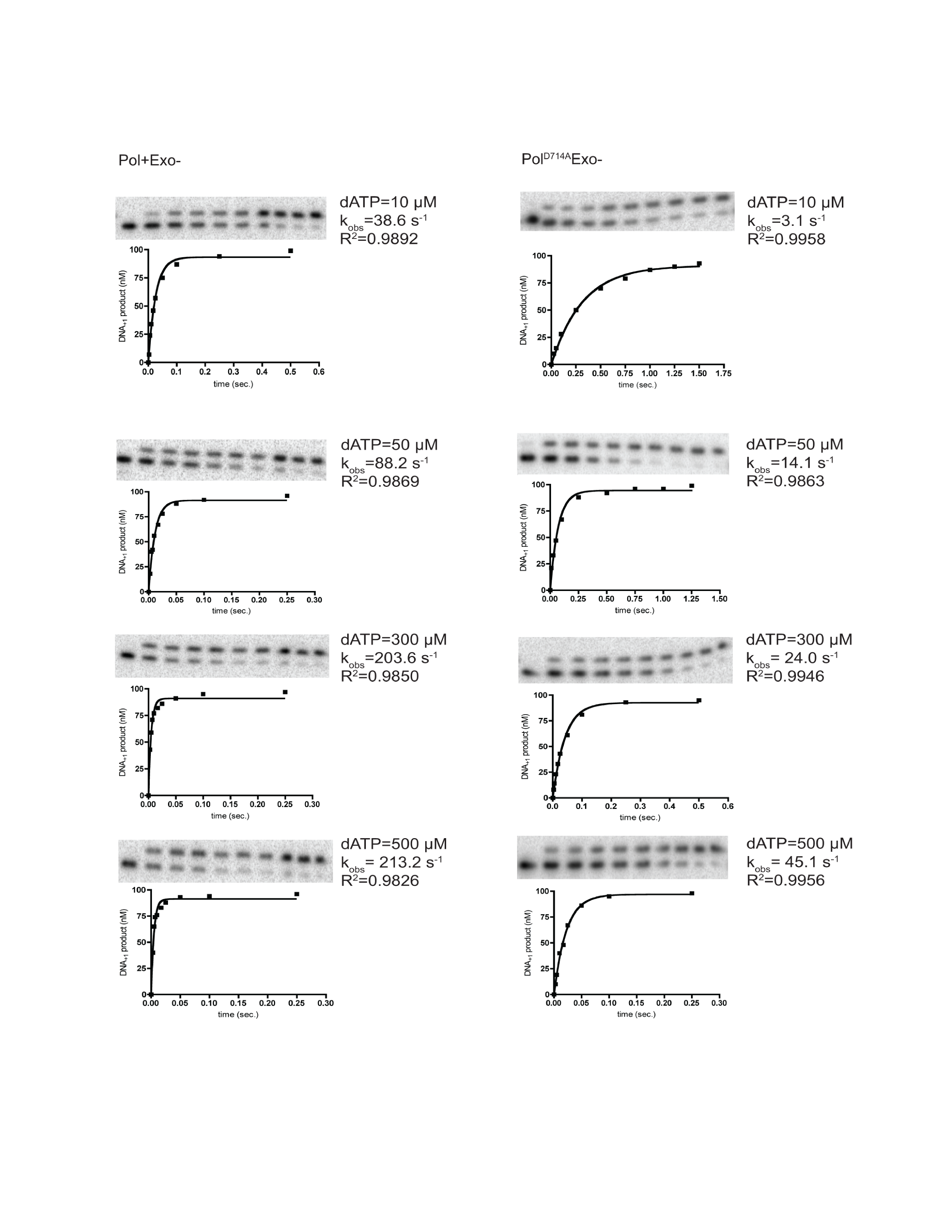

Supplement: Figure S2 — Pre-steady state kinetics of the correct nucleotide incorporation by PolD714A mutant in comparison to the wild type polymerase. Representative gels and the progress curves for four different dATP concentrations (10 µM, 50 µM, 300 µM and 500 µM) are shown. The goodness of the curve fit (R2) and the calculated observed rate constants (kobs) are included for each nucleotide concentration. (TIFF) [file pone.0076700.s002.tiff]

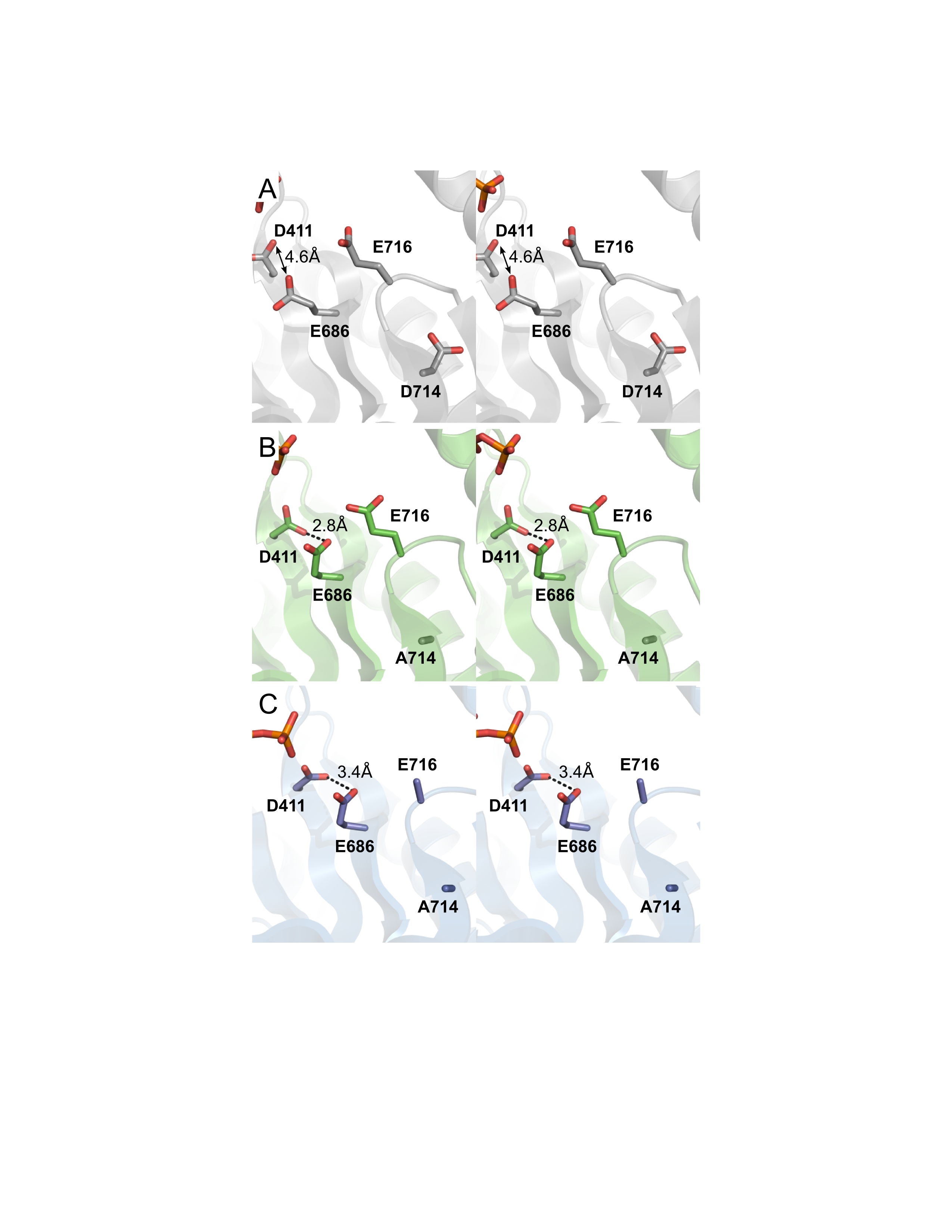

Supplement: Figure S3 — Microenvironment near the A714 residue in wild type and ternary complexes I and II. Stereo images showing the details of the network surrounding residue 714 in the wild type (A), ternary complex I (B) and ternary complex II (C). The distances between D411 and E686 are indicated as an arrow (wild type) and dashed lines (ternary complexes I and II, where they form hydrogen bonds). (TIFF) [file pone.0076700.s003.tiff]

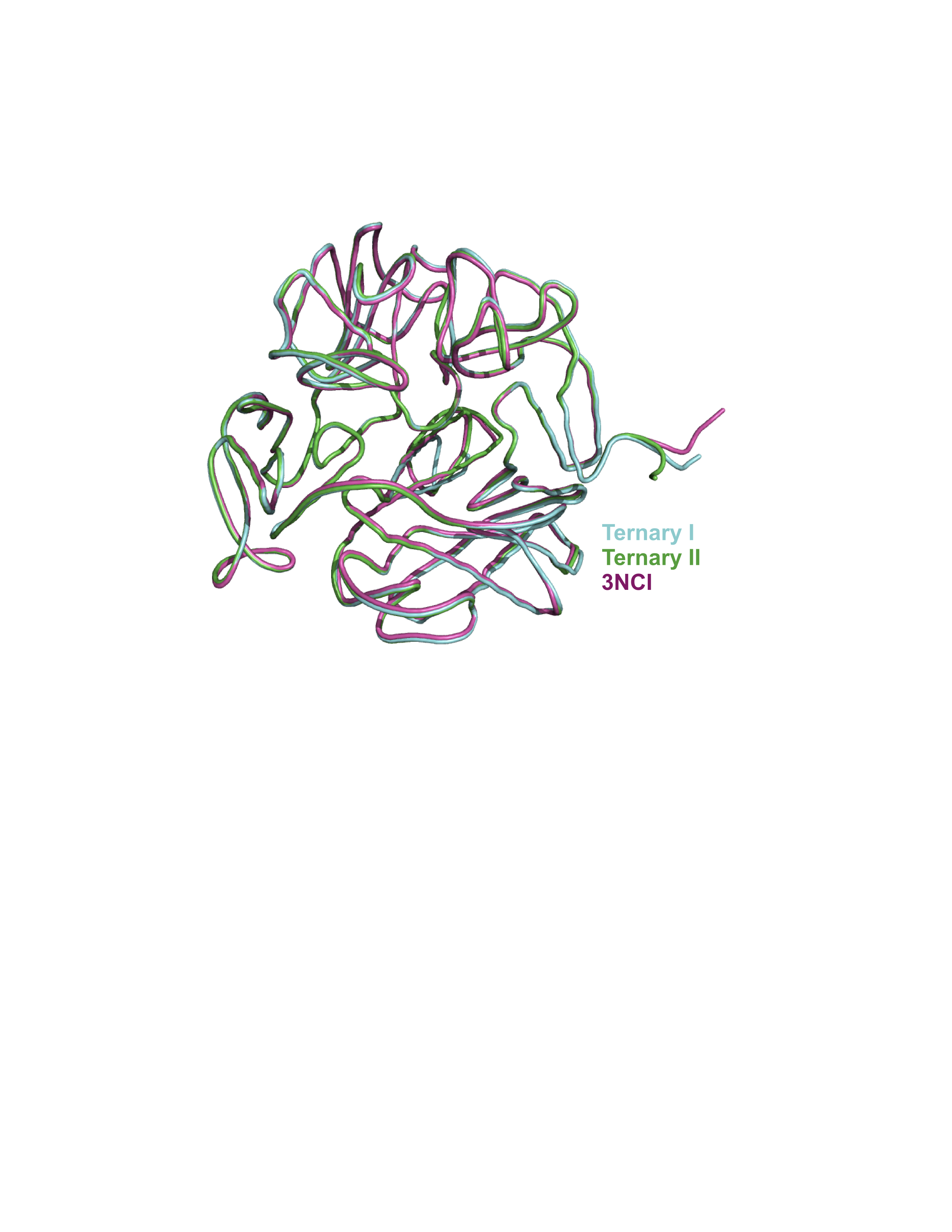

Supplement: Figure S4 — Overlay of the wild type ternary structure with ternary complexes I and II. C-α traces of the wild type (3NCI, violet), ternary complex I (cyan) and ternary complex II (green). The overall fold of the protein is identical in our structures and the wild type structure with RMSD values of 0.6 Å for 763 C-alpha atoms and 0.5 Å for 815 C-alpha atoms for ternary complexes I and II, respectively. (TIFF) [file pone.0076700.s004.tiff]
